# Post-translational modifications in retinoblastoma: mechanisms, immune regulation, and therapeutic opportunities

**DOI:** 10.3389/fimmu.2026.1820785

**Published:** 2026-05-13

**Authors:** Lifei Xu, Bin Wang, Wenwei Li, Xiandan Luo, Lixia Wang, Yi Zheng

**Affiliations:** 1 Department of Ophthalmology, Tongde Hospital of Zhejiang Province, Hangzhou, Zhejiang, China; 2 Department of Urology, The Second Affiliated Hospital of Soochow University, Suzhou, Jiangsu, China; 3 Department of Nursing, Tongde Hospital of Zhejiang Province, Hangzhou, Zhejiang, China

**Keywords:** degradation, PTM, RB, retinoblastoma, ubiquitination

## Abstract

Retinoblastoma is an early-childhood retinal malignancy driven predominantly by biallelic RB1 inactivation and consequent RB–E2F checkpoint deregulation. Post-translational modifications (PTMs) add a rapid, reversible regulatory layer that rewires RB-centered signaling, chromatin control, metabolic adaptation, and therapy resistance. This review summarizes PTM mechanisms relevant to retinoblastoma, highlighting phosphorylation- and ubiquitination-centric circuits, as well as acetylation and methylation that modulate RB pathway function and downstream oncogenic phenotypes. We discuss actionable therapeutic opportunities, including compounds and degraders targeting PTM enzymes, and emphasize underexplored modifications such as SUMOylation, lactylation, and glycosylation that warrant systematic investigation in retinoblastoma. Finally, we integrate PTM biology with emerging immunotherapies and propose rational PTM–immunotherapy combinations and biomarker-guided translation to improve durable eye salvage and metastatic control.

## Introduction

Retinoblastoma is a malignant tumor of the developing retina that almost always presents in early childhood. In 2021, worldwide analyzes counted 57,333 people living with retinoblastoma, alongside 6,274 new diagnoses and 2,762 deaths. On a global, age-standardized basis, the rates per 100,000 population were 0.09 for incidence, and 0.04 for mortality (1). Roughly two in five cases are inherited, stemming from a germline alteration of the RB1 tumor-suppressor gene. Affected children frequently present with tumors in both eyes or multiple foci and remain at elevated lifetime risk for second primary cancers (2). The sporadic form typically features a somatic RB1 mutation limited to the tumor itself, and a minority of cases arise through MYCN amplification in the absence of RB1 inactivation. Retinoblastoma treatment prioritizes survival, then globe and vision preservation, using focal therapies, systemic and eye-directed chemotherapy. Radiotherapy minimized due to secondary cancers, while enucleation remains crucial for unsalvageable eyes (3). Retinoblastoma outcomes differ globally, with >95% survival in high-income versus ~50% in low-income regions due to late diagnosis. Heritable survivors need long-term monitoring for secondary cancers, especially post-radiation (4).

Evidence has suggested that several signaling pathways play a critical role in retinoblastoma tumorigenesis (5). At the core, retinoblastoma is a “RB1–E2F cell-cycle checkpoint” disease, with several important add-on pathways. Most retinoblastomas are initiated by biallelic RB1 inactivation, including germline plus somatic mutations, two independent somatic hits, or promoter hypermethylation–mediated silencing (6, 7). RB1 loss disables pRB-mediated E2F repression, so E2F remains continuously active, driving unchecked G1 to S progression, excessive DNA replication, replication stress, and genomic instability (8). Retinoblastoma arises from maturing cone precursors with high MYCN, mouse double minute 2 homolog (MDM2), S-phase kinase-associated protein 2 (SKP2), whose strong proliferative drive is normally restrained by pRB, while RB1 loss renders them highly tumorigenic (9, 10). Although TP53 remains wild-type, overexpressed MDM2/mouse double minute 4 homolog (MDM4) inactivate p53, enabling RB1-deficient cells under intense oncogenic stress to evade apoptosis and senescence and continue proliferating (11, 12). A small subset of retinoblastomas with wild-type RB1 but high-level MYCN amplification shows MYCN-driven hyperproliferation, metabolic reprogramming and apoptosis resistance, yielding highly aggressive tumors independent of RB1 loss (13). In addition, the phosphoinositide 3-kinase (PI3K), AKT, mechanistic target of rapamycin (mTOR), Wingless/Int-1 (Wnt), β-catenin, Notch, and hedgehog pathways cooperate to drive the initiation and maintenance of malignant cone-derived retinoblastoma (14–17).

Recently, post-translational modification (PTM) has been revealed to contribute to retinoblastoma oncogenesis (18). Common PTMs include phosphorylation, acetylation, methylation, ubiquitination, SUMOylation, glycosylation, palmitoylation, prenylation, nitration, citrullination, ADP-ribosylation, and lactylation (19–22). PTMs are usually dynamic and reversible, allowing cells to rapidly respond to signals such as growth factors, DNA damage, metabolic stress, hypoxia, or immune stimuli (23, 24). Retinoblastoma tumor suppressor protein (pRB) has been reported to have various PTMs in numerous diseases, including phosphorylation, SUMOylation, methylation, ubiquitination (25, 26). A distinct subset of unilateral retinoblastomas lacks RB1 mutations yet harbors high MYCN amplification and abundant phosphorylated (inactive) pRB, indicating that RB1 gene loss or pRB hyperphosphorylation is requisite for tumor initiation independent of MYCN status (27). In this study, we will describe the role of PTMs, especially for pRB, in retinoblastoma oncogenesis and progression.

## PTMs in retinoblastoma

### Phosphorylation in retinoblastoma

**Figure 1:**
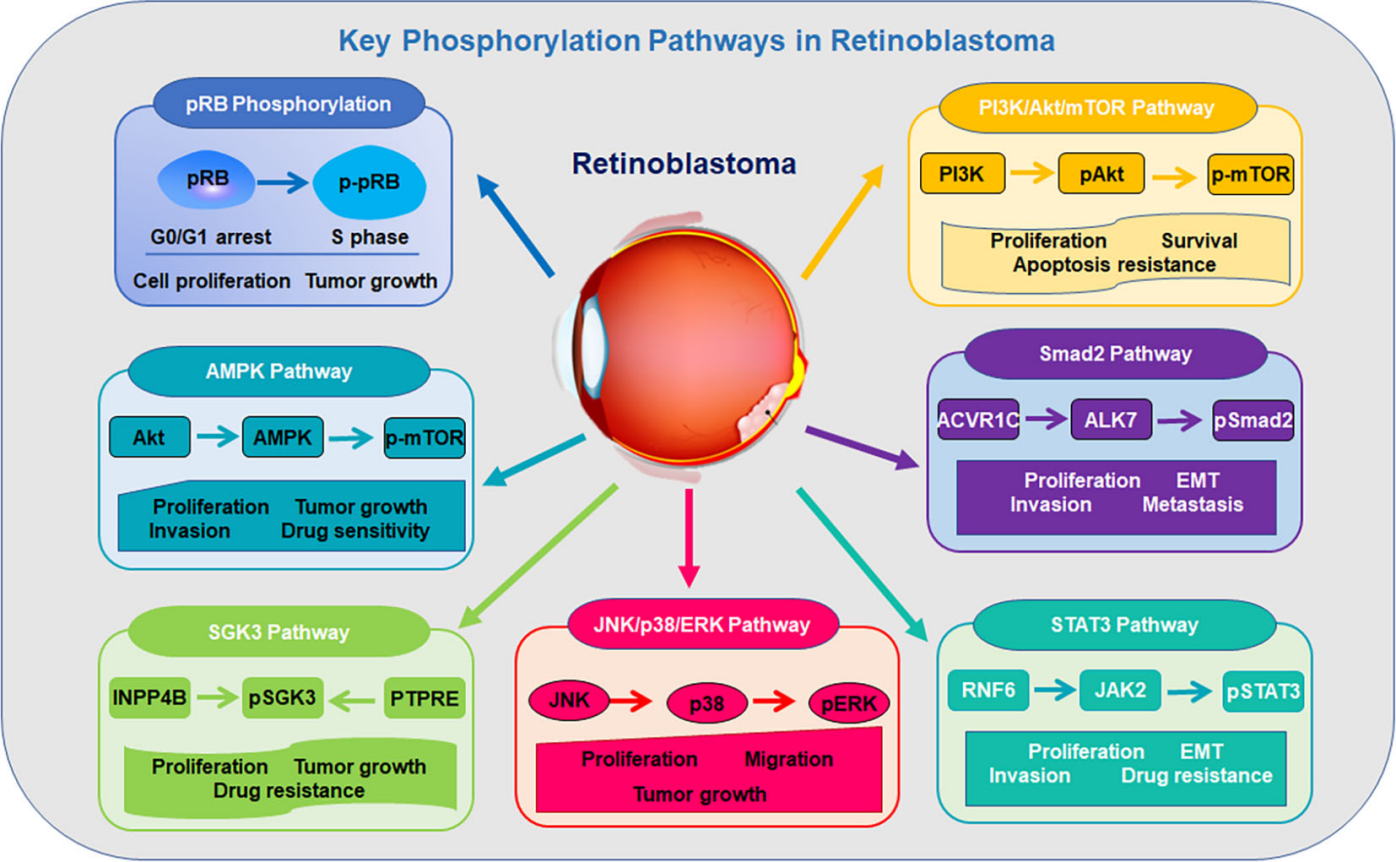
Key phosphorylation pathways in retinoblastoma progression.

Protein phosphorylation is a reversible PTM where kinases add phosphate groups, typically to serine, threonine, or tyrosine (28). Phosphorylation has been identified to alter protein activity, interactions, and signaling pathways (29). In the following paragraphs, we describe the role of phosphorylation in retinoblastoma tumorigenesis and progression (**Figure 1**).

#### pRB phosphorylation

Exogenous pRB suppresses tumorigenicity, and its function is regulated by reversible phosphorylation. pRB is highly phosphorylated in proliferating cells, especially in S phase, but dephosphorylated in resting and differentiating cells (30). Similarly, another study showed that pRB1 is underphosphorylated in G0/G1 but becomes multisite-phosphorylated at the G1/S boundary and in S phase (31). Overexpressed cyclins A and E bypass pRB-mediated growth arrest in pRB-deficient SAOS-2 cells by driving essential pRB hyperphosphorylation (32). Transforming growth factor beta (TGF-β1) added in mid-late G1 blocks the scheduled phosphorylation of pRB to keep it underphosphorylated and arrest cells in late G1 (33). Orthodenticle homeobox 2 (OTX2) is frequently amplified/overexpressed in retinoblastoma, and its genetic or ATRA-mediated suppression reduces C-MYC/cone-rod homeobox (CRX) expression, curtails proliferation and tumor growth, and increases pRB phosphorylation (34). Together, these findings highlight reversible pRB phosphorylation as a central regulatory mechanism in retinoblastoma-related cell-cycle control and tumor progression.

#### PI3K/Akt/mTOR phosphorylation

Protein arginine deiminase II (PADI2) is overexpressed in retinoblastoma via E2F, and its inhibition lowers phosphorylated AKT, increases cleaved poly(ADP-ribose) polymerase (PARP), triggers apoptosis, shrinks tumors in retinoblastoma (35). In retinoblastoma, ADAM metallopeptidase domain 17 (ADAM17) depletion reduces AKT phosphorylation and inhibits the tumorigenic and migration of retinoblastoma cells (36). LncRNA urothelial carcinoma associated 1 (UCA1) is upregulated in retinoblastoma and correlates with larger tumors, optic nerve invasion, higher grade, where it drives proliferation and cell-cycle progression and suppresses apoptosis by activating the PI3K and Akt phosphorylation (37). LncRNA antisense non-coding RNA in the INK4 locus (ANRIL) acts as a miR-328-3p sponge to upregulate tuberous sclerosis complex 1 (TSC1) and UNC-51 like autophagy activating kinase 2 (ULK2), suppress mTOR phosphorylation, activate autophagy, and thereby promote Y79 retinoblastoma cell proliferation and cisplatin resistance (38). Together, dysregulated PI3K/Akt/mTOR phosphorylation plays a critical role in retinoblastoma progression, survival, and therapeutic resistance.

#### JNK/p38/ERK phosphorylation

Evidence reveals that miR-889-3p is upregulated in retinoblastoma and directly targets bone morphogenetic protein receptor type 2 (BMPR2) to activate the c-Jun N-terminal kinase (JNK)/p38 mitogen-activated protein kinase (MAPK)/extracellular signal-regulated kinase (ERK) pathway via increased phosphorylation, which promotes proliferation, migration, and tumor growth (39). Quercetin suppresses Y79 retinoblastoma cells by inducing G1 arrest, triggering mitochondrial depolarization and caspase-9/3–dependent apoptosis via phosphorylation of JNK and p38 MAPK (40). C-MET is overexpressed in Y79 retinoblastoma and drives proliferation and tumor growth via hepatocyte growth factor (HGF)-triggered ERK1/2 phosphorylation that promotes pyruvate kinase M2 (PKM2) nuclear translocation, PKM2–histone H3–dependent upregulation of cyclin D1 and c-Myc (41). Hence, aberrant JNK/p38/ERK phosphorylation contributes substantially to retinoblastoma progression and survival.

#### SGK3 phosphorylation

Serum/glucocorticoid regulated kinase 3 (SGK3) is a serine/threonine kinase in the SGK family, AKT-related, acting downstream of PI3K to regulate proliferation, survival, and migration. SGK3 can also drive AKT-independent tumorigenesis and crosstalk with RAS/rapidly accelerated fibrosarcoma (RAF)/ERK (42). In retinoblastoma, inositol polyphosphate-4-phosphatase type II B (INPP4B) is reduced in etoposide-resistant cells and overexpression of INPP4B suppresses growth and induces apoptosis via increasing SGK3 phosphorylation, implicating SGK3 and possible glycogen synthase kinase 3 (GSK3)-related signaling (43). Protein tyrosine phosphatase receptor type E (PTPRE) is upregulated in etoposide-resistant retinoblastoma, where loss of miR-631 permits its expression. PTPRE knockdown or miR-631 restoration curtails proliferation and tumor growth, resensitizes cells to etoposide, increases caspase-dependent apoptosis, and alters phosphorylation of SGK3 (44). Collectively, dysregulated SGK3 phosphorylation is involved in retinoblastoma progression and etoposide resistance.

#### AMPK phosphorylation

AMP-activated protein kinase (AMPK) is an energy-sensing serine/threonine kinase that restores energy balance by promoting catabolism, inhibits anabolism, and regulates metabolism, autophagy, and growth (45). Atovaquone inhibits retinoblastoma cell growth, increases chemosensitivity, exploits higher mitochondrial activity to induce oxidative damage, and modulates Akt/AMPK/mTOR via mitochondrial dysfunction (46). Salinomycin suppresses retinoblastoma growth, targets stem-like invasive cells, and inhibits xenograft tumors by disrupting mitochondrial respiration, causing oxidative stress, activating AMPK, and inhibiting mTOR (47). Loss of HK1 drives retinoblastoma tumors toward oxidative phosphorylation (OXPHOS), whereas restoring hexokinase 1 (HK1) or RB1 shifts cells back to glycolysis via cytoplasmic HK1 binding LKB1 to phosphorylate AMPKα at Thr172, thereby suppressing proliferation and invasion, sensitizing to chemotherapy, and reducing tumor burden *in vivo* (48). Taken together, AMPK phosphorylation is a key metabolic regulatory event in retinoblastoma.

#### STAT3 phosphorylation

Signal transducer and activator of transcription 3 (STAT3) is a cytokine-activated transcription factor phosphorylated downstream of JAK kinases that dimerizes, enters the nucleus, and drives genes promoting proliferation and survival (49). In retinoblastoma, integrin subunit alpha 1 (ITGA1)/integrin subunit beta 1 (ITGB1) are overexpressed and promote tumor growth by activating STAT3, while ITGA1 inhibition suppresses proliferation, migration, and xenograft progression (50). RNF6 is upregulated in carboplatin-resistant RB cells and drives multi-drug resistance by activating Janus kinase 2 (JAK2)/STAT3 signaling (51). One study showed that miR-452-5p is upregulated in retinoblastoma and correlates with tumor size and differentiation. Moreover, miR-452-5p knockdown inhibits proliferation, invasion and epithelial-mesenchymal transition (EMT), promotes apoptosis, by relieving suppressors of cytokine signaling (SOCS3) suppression and reducing phosphorylation of JAK2 and STAT3 (52). LncRNA H19 is downregulated in retinoblastoma and functions as a tumor suppressor by sponging the miR-17–92 cluster to upregulate p21 and inhibit STAT3 phosphorylation, leading to induction of cell-cycle arrest and apoptosis (53). In a word, aberrant STAT3 phosphorylation is closely associated with retinoblastoma growth, drug resistance, and malignant progression.

#### SMAD2 phosphorylation

SMAD family member 2 (Smad2) is a TGF-β/Activin/Nodal–responsive transcription factor that is activated by receptor phosphorylation to regulate genes controlling EMT, invasion, and cell survival (54). In invasive retinoblastoma, activin A receptor type 1C (ACVR1C)/activin receptor-like kinase 7 (ALK7) activates smad2 signaling, and blocking ACVR1C or smad2 suppresses invasion, tumor growth, EMT markers, and metastatic dissemination *in vivo* (55). LncRNA ILF3 antisense RNA 1 (ILF3-AS1) is upregulated in retinoblastoma and promotes proliferation, invasion, and tumor growth by sponging miR-132-3p and releasing Smad2 (56). LncRNA SND1 intronic transcript 1 (SND1-IT1) is overexpressed in retinoblastoma, predicts poor prognosis, and promotes proliferation, migration, invasion, and tumorigenesis by sponging miR-132-3p to upregulate Smad2 (57). Circ_0000527 is upregulated in retinoblastoma and promotes proliferation, invasion, angiogenesis, and tumor growth by sponging miR-1236-3p to derepress Smad2 signaling (58). Nodal drives retinoblastoma growth, proliferation, and invasion by increased Smad2 phosphorylation in WERI Rb1 and Y79 retinoblastoma cells (59). Therefore, aberrant SMAD2 phosphorylation is an important driver of retinoblastoma progression, invasion, and metastatic potential.

### Ubiquitination in retinoblastoma

**Figure 2:**
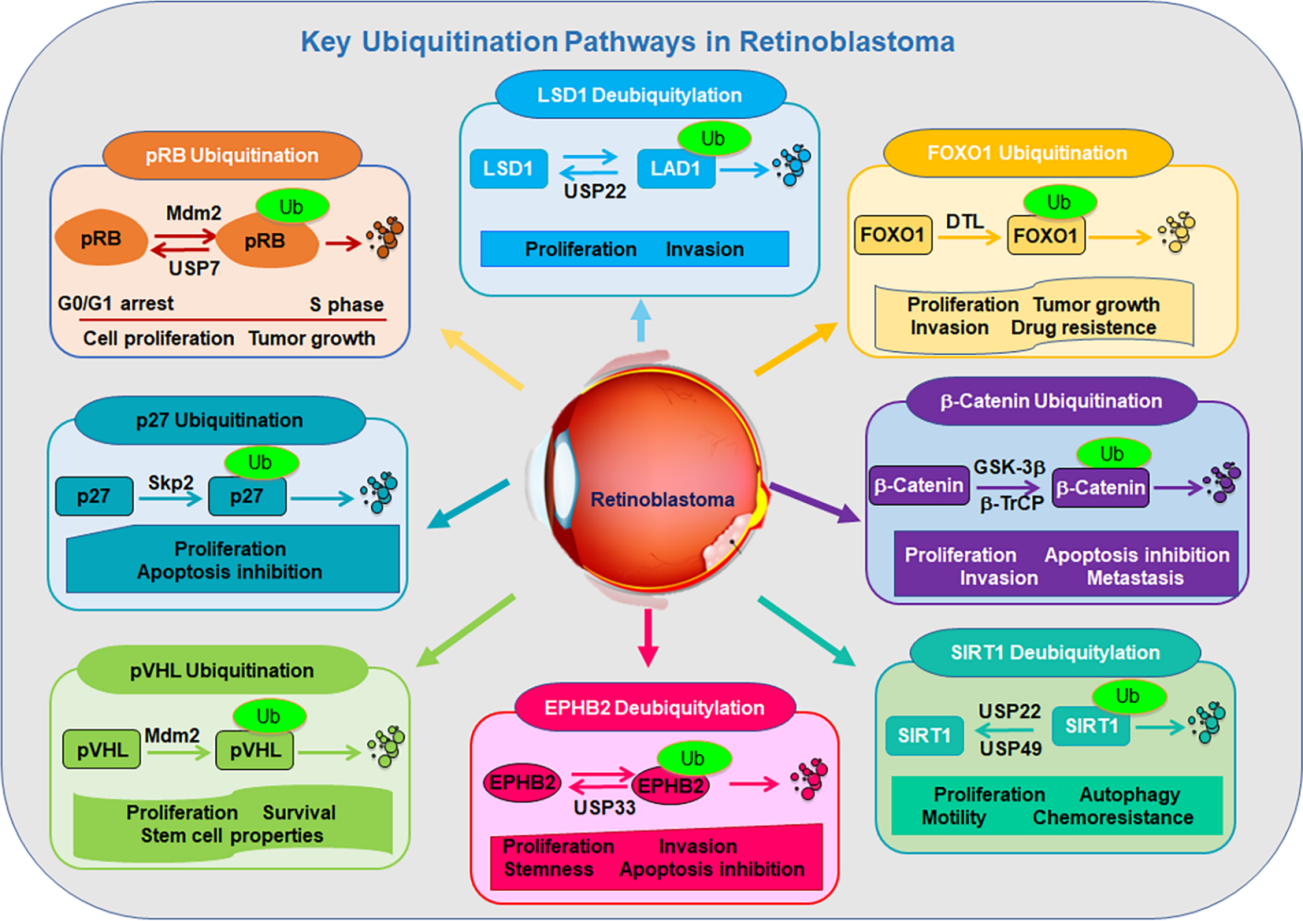
Key ubiquitination pathways in retinoblastoma progression.

Ubiquitination is a common PTM in which E1/E2/E3 enzymes attach ubiquitin to target proteins to control their degradation and localization (60). E1 activates ubiquitin in ATP-dependent manner, E2 carries it as a thioester conjugate, and E3 ligases recognize substrates and catalyze ubiquitin transfer to specific target proteins (61, 62). Integrating transcriptomics with cellular validation, Chen et al. reported that retinoblastoma progression is associated with dysregulated protein ubiquitination and highlighted eight downregulated ubiquitination genes, such as ubiquitin-conjugating enzyme E2 E1 (UBE2E1), S-phase kinase-associated protein 1 (SKP1), F-box protein 9 (FBXO9), F-box protein 15 (FBXO15), and ring finger protein 14 (RNF14) (63). In the next section, we discuss the function of protein ubiquitination in retinoblastoma (**Figure 2**).

#### Ubiquitination in retinoblastoma disease

##### p27 ubiquitination

p27 is a cyclin-dependent kinase inhibitor that restrains G1–S cell-cycle progression, and its loss promotes uncontrolled proliferation (64). In retinoblastoma, T187-phospho-p27 appears in prophase/metaphase and co-localizes with nuclear cdc2, implying p27–cdc2 interaction during M-phase (65, 66). S-phase kinase–associated protein 2 (Skp2) is an SCF E3 ubiquitin ligase F-box subunit that drives cell-cycle progression by targeting p27 and other regulators for ubiquitin-mediated degradation (67). In retinoblastoma, skp2 is overexpressed and promotes proliferation (68). In Rb1-deficient contexts, Skp2 becomes an essential survival factor because Skp2 inactivation prevents spontaneous tumorigenesis in Rb1^+/−^ mice by stabilizing p27, triggering apoptosis of RB1-deficient human retinoblastoma cells (69). Together, these findings indicate that aberrant p27 ubiquitination, particularly through the Skp2 axis, plays a critical role in retinoblastoma cell survival and tumor development.

##### pVHL ubiquitination

Von Hippel–Lindau protein (VHL) is a tumor-suppressor E3 ubiquitin ligase component that targets hypoxia-inducible factor 1 alpha (HIF1-α) for oxygen-dependent degradation, thereby limiting hypoxia-driven angiogenesis and growth programs (70). In retinoblastoma, miR-222 promotes vincristine resistance by suppressing VHL, stabilizing HIF1α (71). MDM2 is an E3 ubiquitin ligase that negatively regulates p53 by binding and ubiquitinating it, promoting p53 degradation and dampening DNA-damage–induced cell-cycle arrest and apoptosis (72). In retinoblastoma, high MDM2 is essential for proliferation and xenografts via p53-independent MYCN upregulation, sustaining cone-precursor tumor circuitry (73). MDM2 sustains retinoblastoma survival under hypoxia by binding to pVHL to ubiquitinate and degrade it, thereby elevating HIF-1α. Inhibiting MDM2 and/or HIF-1α kills RB cells and diminishes stem-like properties (74). Taken together, dysregulated pVHL ubiquitination contributes to hypoxia adaptation, stemness, and chemoresistance in retinoblastoma.

##### FOXO1 ubiquitination

Forkhead box O1 (FOXO1) is a forkhead transcription factor that integrates PI3K/AKT and stress signals to regulate genes controlling cell-cycle arrest, apoptosis, and metabolism (75). Camptothecin induces apoptosis in Y79 retinoblastoma cells by activating foxo1 by its dephosphorylation and increased transcriptional activity, upregulating Bim (76). Enhancer of zeste 2 (EZH2) is highly expressed in retinoblastoma and predicts poor survival. Inhibition of EZH2 suppress proliferation, metastasis, and glycolysis via STAT3/FoxO1 (77). Denticleless E3 ubiquitin protein ligase homolog (DTL), also known as chromatin licensing and DNA replication factor 2 (CDT2), is the substrate receptor of the CRL4^Cdt2^ E3 ubiquitin ligase. DTL recognizes PCNA-bound proteins and targets factors like chromatin licensing and DNA replication factor 1 (CDT1), p21, and SET8 for proteasomal degradation (78). Carboplatin-resistant retinoblastoma suppresses nuclear FOXO1 via DTL-driven ubiquitination and proteasomal degradation, and restoring FOXO1 by DTL knockdown, especially combined with LOM612, resensitizes cells, curbing proliferation, invasion, and tumor growth (79). Hence, FOXO1 ubiquitination contributes to retinoblastoma progression and chemoresistance by disabling a key tumor-suppressive transcriptional program.

##### β-catenin ubiquitination

β-catenin accumulates in WNT signaling pathway to drive TCF/LEF-dependent transcriptional programs and promote proliferation and differentiation (80). S-adenosylmethionine (SAM) suppresses Y79 retinoblastoma proliferation, induces G1 arrest and apoptosis, alters morphology, and acts through inhibiting the Wnt2/β-catenin pathway (81). LncRNA maternally expressed 3 (MEG3) is downregulated in retinoblastoma and its overexpression suppresses proliferation and migration, increases apoptosis, reduces xenograft growth, by inhibiting PI3K/Akt/mTOR signaling (82). Moreover, low lncRNA MEG3 expression in retinoblastoma correlates with invasiveness, while MEG3 suppresses migration and metastasis by promoting β-catenin–GSK-3β binding to drive β-catenin phosphorylation, ubiquitination, and degradation, resulting in inactivation of Wnt signaling (83). Collectively, impaired β-catenin ubiquitination contributes to aberrant Wnt signaling and retinoblastoma progression.

#### Deubiquitination in retinoblastoma disease

##### USP22 deubiquitinates SIRT1 and LSD1

SIRT1 is an NAD^+^-dependent deacetylase that modulates chromatin and key regulators such as p53 and FOXO proteins (84, 85). LncRNA KCNQ1 antisense transcript 1 (KCNQ1OT1) is upregulated in RB and sponges miR-124 to derepress SP1, modulating SIRT1/JNK signaling (86). USP22 promotes retinoblastoma cell proliferation and telomerase activity by increasing TERT and suppressing p53, thereby reducing senescence, apoptosis, and DNA damage (87). In retinoblastoma, phosphorylated p38 MAPK suppresses USP22, diminishing its deubiquitination-mediated stabilization of SIRT1, which elevates sclerostin (SOST) and collectively inhibits proliferation, migration, invasion, and tumor growth (88). In retinoblastoma, miR-362-3p is reduced while USP22/lysine-specific demethylase 1 (LSD1) increase. Moreover, USP22 depletion suppresses growth and invasion by reducing LSD1 deubiquitination (89). In summary, USP22-mediated deubiquitination of SIRT1 and LSD1 contributes to retinoblastoma progression by sustaining oncogenic signaling and malignant phenotypes.

##### USP33 deubiquitinates EPHB2

In retinoblastoma, USP33 is upregulated and promotes cell growth and tumorigenesis while suppressing apoptosis and G1 arrest by activating the SP1/PI3K/AKT pathway (90). EPH receptor B2 (EPHB2) is a receptor tyrosine kinase of the Eph family that binds ephrin-B ligands to regulate cell–cell signaling, migration, and adhesion, often influencing tumor invasion and metastasis (91). LINC00488 is upregulated in RB and sponges miR-30a-5p to increase EPHB2, promoting Y79 cell malignancy (92). In retinoblastoma, EPHB2 is upregulated and portends poorer survival, while USP33 deubiquitinates and stabilizes EPHB2 to activate Wnt/β-catenin signaling, thereby enhancing proliferation, invasion, and stemness and suppressing apoptosis (93). Thus, the USP33–EPHB2 deubiquitination axis promotes retinoblastoma progression.

##### USP49 deubiquitinates SIRT1

USP49 has been implicated in tumorigenesis and cancer progression (94). SIRT1 is broadly expressed in retinoblastoma; however, its expression level is not associated with high-risk histopathological features or patient survival outcomes (95). In retinoblastoma, USP49 is upregulated and drives carboplatin resistance by activating autophagy, which is upregulated via an insulin-like growth factor 2 mRNA-binding protein 3 (IGF2BP3)-mediated m^6^A-dependent mechanism. Mechanistically, USP49 stabilizes SIRT1 by preventing its ubiquitination, leading to promotion of tumor growth and chemoresistance (96). These findings suggest that the USP49–SIRT1 axis may represent a potential therapeutic target for overcoming chemoresistance in retinoblastoma.

#### Acetylation in retinoblastoma

**Figure 3:**
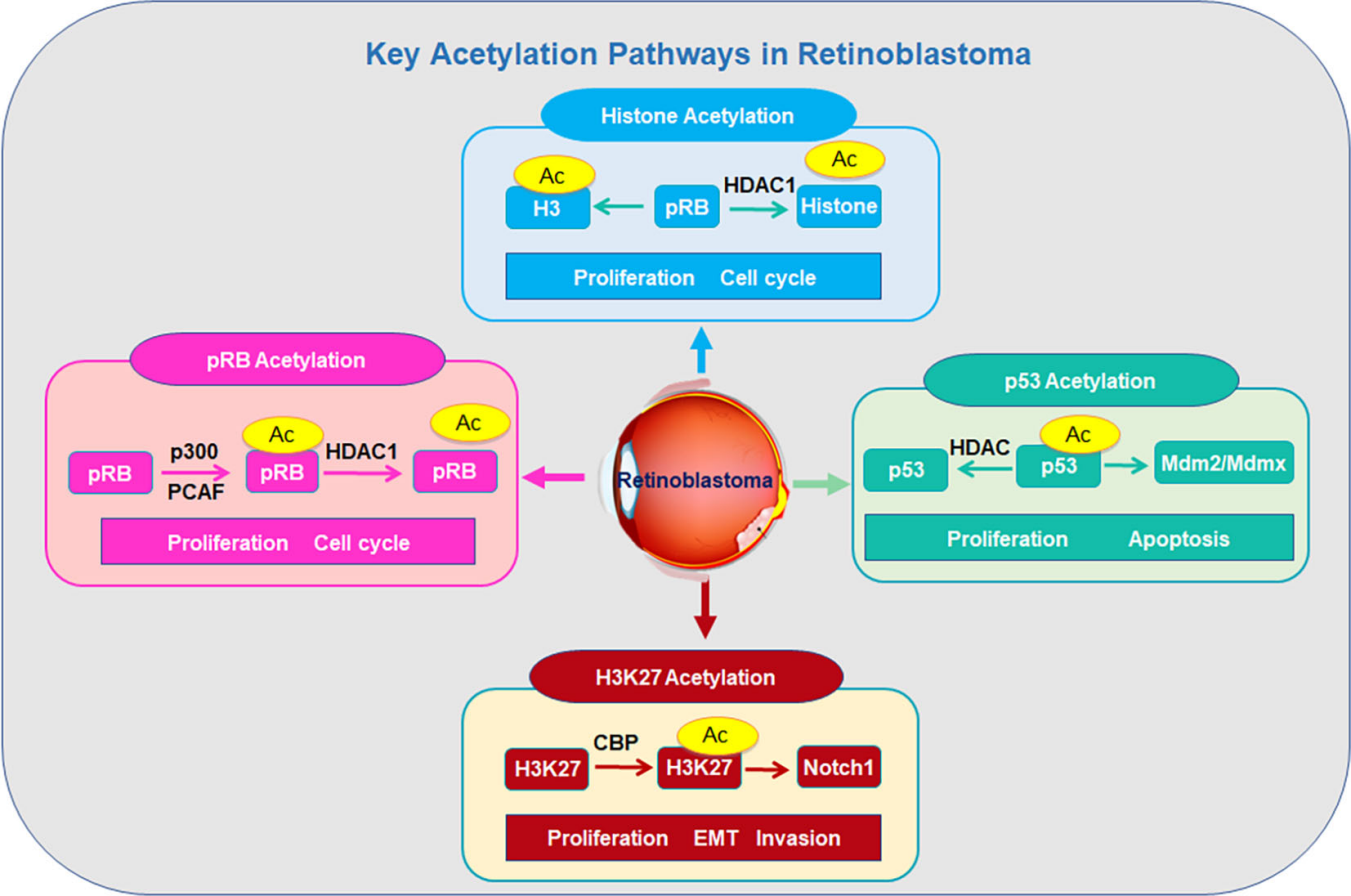
Key acetylation pathways in retinoblastoma progression.

Protein acetylation is a reversible PTM in which acetyltransferases add acetyl groups to lysine residues, which modulates protein function (97). Protein acetylation is written by acetyltransferases (KATs/NATs) using acetyl-CoA and erased by deacetylases (HDACs and sirtuins) (98). Acetylation has been discovered to play a critical role in retinoblastoma development and progression (**Figure 3**).

##### RB acetylation

Several studies have shown the RB acetylation in various conditions. SIRT1 binds the RB pocket and NAD-dependently deacetylates RB to favor RB inactivation alongside hyperphosphorylation, while cell arrest increases RB acetylation and decreases phosphorylation (99). RB is monomethylated by SET and MYND domain containing 2 (SMYD2) at lysine 860, and is involved in cell cycle, differentiation, and DNA damage response (100). During differentiation, pRB is acetylated by p300/CBP-associated factor (P/CAF), which is required for terminal cell-cycle exit and induction of late myogenic genes (101). DNA damage triggers acetylation of pRB at K873/874, which hinders pRB phosphorylation, keeps pRB growth-suppressive (102). During keratinocyte differentiation, PCAF-mediated acetylation of RB is required to keep RB nuclear and enforce cell-cycle exit (103). High-risk HPV16 E7 dimerizes to bridge CBP/p300 (TAZ2) and pRB into a ternary complex, thereby promoting pRB acetylation and disrupting cell-cycle control (104). E1A recruits p300/CBP to acetylate pRB, hinders Cdk-mediated phosphorylation, and strengthens pRb–MDM2 binding (105). Thus, RB acetylation is an important regulatory modification that influences RB activity in cell-cycle control, differentiation, and stress responses.

##### RB regulates histone acetylation

pRB represses the cyclin E promoter by recruiting histone deacetylase 1 (HDAC1) to a specific nucleosome, reducing its histone acetylation and altering its conformation (106). RB family proteins safeguard constitutive heterochromatin by partnering with the H4K20 methyltransferases Suv4-20h1/2 to maintain H4K20 trimethylation. Loss of RB/RB transcriptional corepressor like 1 (RBL1)/RBL2 causes genomic instability with reduced DNA methylation, increased H3 acetylation, and diminished H4K20me3 at pericentric/telomeric regions (107). RB uses its pocket domain to recruit HDAC1 to E2F-target promoters, enforcing chromatin repression via histone deacetylation, supporting acetylation control in cell-cycle regulation (108, 109). Therefore, RB not only undergoes acetylation-dependent regulation itself but also actively shapes the histone acetylation landscape to maintain chromatin stability and suppress inappropriate cell-cycle progression.

##### HDAC in retinoblastoma

HDACs remove acetyl groups from lysine residues on histone and non-histone proteins. Dysregulated HDAC activity in retinoblastoma perturbs acetylation of key tumor suppressors/oncogenes and intersects with the RB pathway to skew cell cycle control, apoptosis, and the tumor microenvironment (110). HDAC9 is overexpressed in retinoblastoma and associated with poor prognosis, and its knockdown reduces cyclin E2/CDK2, induces G1 arrest, suppresses proliferation, and inhibits tumor growth *in vivo* (111). In addition, miR-101-3p is downregulated in retinoblastoma, and its overexpression suppresses WERI-Rb-1/Y79 cell viability and cell-cycle progression by directly targeting the EZH2 and HDAC9 (112). These studies indicate that protein acetylation could be involved in retinoblastoma progression.

##### p53 and H3K27 acetylation

Evidence has suggested that p53 acetylation is critical in cancer development and progression (113, 114). Low-dose paclitaxel (5 nM) triggers G2/M arrest and robust E2F1 upregulation in Y79 retinoblastoma cells, activating p14^ARF^-mediated p53 stabilization by phosphorylation and acetylation (115). HDAC inhibitors valproic acid (VPA) and FK228 sensitize Y79 and WERI-Rb1 cells to radiation by boosting p53 acetylation at Lys382 and Ser46 phosphorylation, weakening MDM2/MDMX binding, amplifying DNA-damage signaling, and driving caspase-3–mediated apoptosis (116). In retinoblastoma, CBP-mediated H3K27 acetylation upregulates lincRNA regulator of reprogramming (ROR), which sponges miR-32-5p to activate Notch signaling and drive EMT, promoting invasion, metastasis, and recurrence *in vitro* and *in vivo* (117).

#### Methylation in retinoblastoma

**Figure 4:**
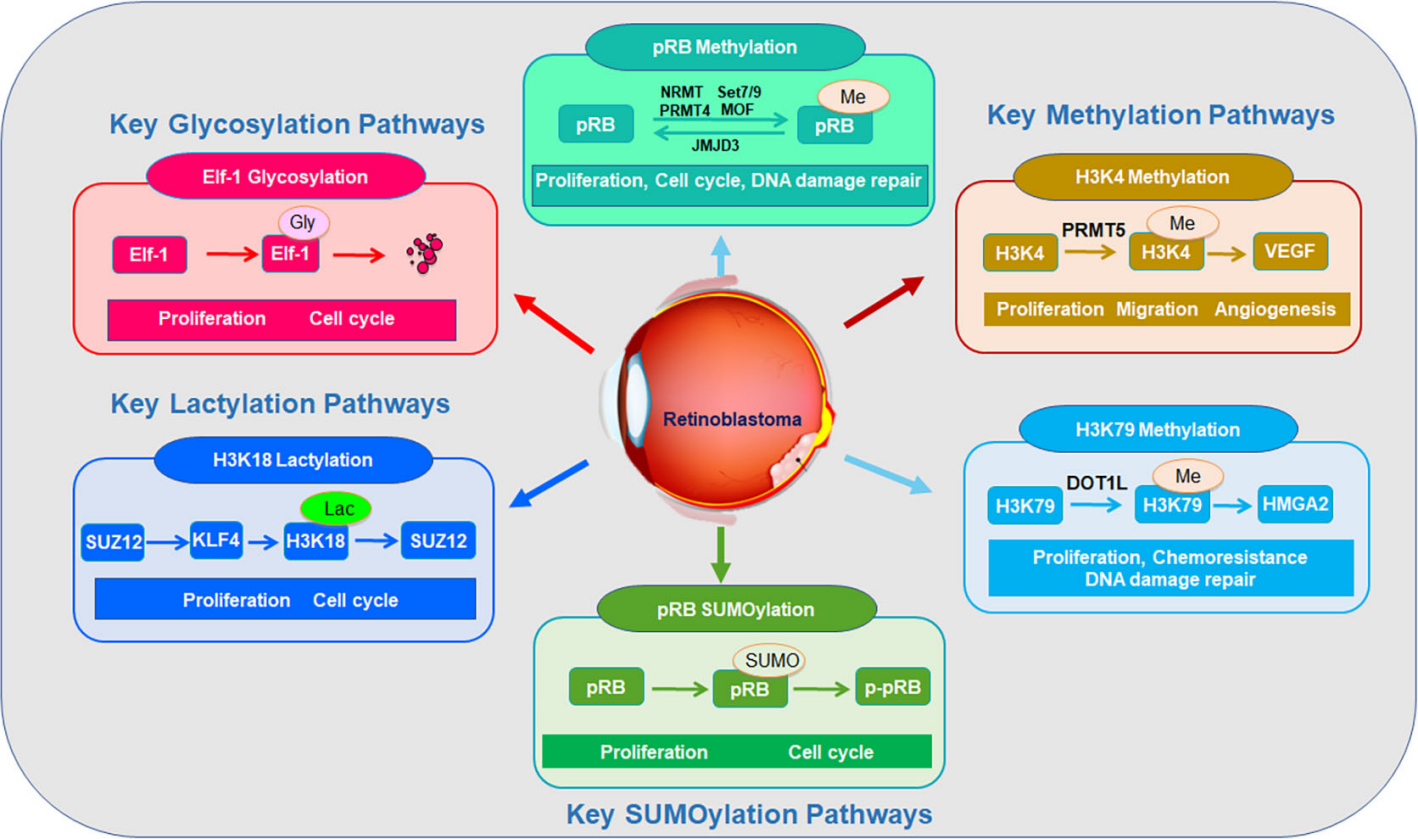
Key methylation, SUMOylation, glycosylation pathways in retinoblastoma progression.

Protein methylation is a reversible PTM in which methyltransferases add one to three methyl groups to lysine or arginine residues. Protein methylation is installed by lysine/arginine methyltransferases (KMTs/PRMTs) using S-adenosyl-L-methionine (SAM) and removed by demethylases, such as KDMs/LSD1. Protein methylation has been reported to participate into retinoblastoma development and progression (**Figure 4**).

##### Rb methylation

pRB is essential not only for myoblast differentiation but for maintaining permanent cell-cycle exit in myotubes by preserving H3K27 trimethylation on cell-cycle genes (118). Researchers identified N-terminal RCC1 methyltransferase (NRMT) as the first α-N-methyltransferase that recognizes an N-terminal Met-(Ala/Pro/Ser)-Pro-Lys motif to catalyze α-N-methylation of proteins including RCC1, SET, and RB, and showed that NRMT knockdown disrupts bipolar spindle formation and chromosome segregation (119). Set7/9 methylates pRB at K810 within the SPXK Cdk-recognition motif, blocking Cdk binding and subsequent serine phosphorylation to keep pRB in a hypophosphorylated, growth-suppressive state (120). PRMT4/CARM1 arginine-methylates pRB and this methylation facilitates Cdk-driven phosphorylation, disrupts E2F1/DP1–pRB complex formation, and thereby weakens pRB’s growth-suppressive repression of E2F (121). Mono-methylation of pRB at K810 recruits the PHF20L1 reader to E2F target genes, bringing in the MOF acetyltransferase complex, to reinforce a pRB-dependent G1–S checkpoint (122). K810 methylation on pRB is specifically read by 53BP1’s tandem Tudor domain, enabling 53BP1 recruitment to E2F target genes and integrating pRb’s hypophosphorylated, growth-suppressive activity with the DNA-damage response (123). JMJD3, upregulated during Ras^V12^-induced senescence, demethylates pRB at K810, which disrupts CDK4 binding and lowers pRB phosphorylation at S807/811 to promote senescence-associated heterochromatin foci (SAHF) formation (124).

##### Histone methyltransferase DOT1L

Histone methyltransferase disruptor of telomeric silencing 1-like (DOT1L) is a conserved, non-SET domain enzyme that catalyzes mono-, di-, and tri-methylation of H3K79 within the nucleosome core, thereby regulating transcriptional programs (125). DOT1L has been identified as a pivotal regulator in cell cycle progression (126). DOT1L promotes transcription, DNA repair, and cell-cycle programs and sustains homeobox A cluster (HOXA)/Meis homeobox 1 (MEIS1) in mixed-lineage leukemia (MLL) leukemia and drives EMT/angiogenesis in solid tumors (127). DOT1L inhibition chemosensitizes retinoblastoma by impairing early DNA-damage responses and downregulating high mobility group AT-hook 2 (HMGA2) via inhibiting H3K79 methylation at the HMGA2 promoter, thereby reducing CHK1 phosphorylation and markedly enhancing etoposide efficacy in orthotopic models (128). Hence, DOT1L is involved in chemoresistance in retinoblastoma.

##### Arginine methyltransferase PRMT5

Protein arginine methyltransferase 5 (PRMT5) is a type II protein arginine methyltransferase that symmetrically dimethylates histones and splicing factors, driving transcriptional repression (129). PRMT5 symmetrically dimethylates arginine on histone and non-histone proteins, regulating transcription, splicing, DNA repair, and apoptosis, leading to tumorigenesis (130, 131). PRMT5 is overexpressed in retinoblastoma and drives growth. Its inhibitor EPZ015666 suppresses proliferation, induces G1 arrest via p53–p21/p27–CDK2, and slows xenograft tumors (132). In retinoblastoma, PRMT5 drives VEGFA-dependent proliferation, angiogenesis, and migration by promoting H3K4me3 at the vascular endothelial growth factor A (VEGFA) promoter, while PRMT5 overexpression lowers matrix metallopeptidase (MMP) 1/2/9 and suppresses tumor growth (133). Collectively, PRMT5 promotes retinoblastoma proliferation and migration.

#### SUMOylation in retinoblastoma

SUMOylation is a reversible PTM in which SUMO proteins are covalently attached to lysine residues on target proteins to regulate their localization and stability (134). One study revealed that endogenous RB is SUMOylated in early G1 in human cells (135). Another study showed that pRB is SUMOylated at K720 within its pocket domain, preferentially in the active, hypophosphorylated state, and that viral/cellular LxCxE-motif interactions suppress this modification, tumor-type pocket mutations abolish it. Loss of SUMOylation slightly increases pRB’s E2F-repressive activity (136). During early G1, RB is SUMOylated, which recruits SIM-containing CDK2 to drive RB hyperphosphorylation, release E2F-1, and promote proliferation (137). SENP1-modulated SUMO1 conjugation of RB and Lamin A/C enables their interaction, forming a nuclear complex that protects both proteins from proteasomal degradation and thereby regulates RB turnover (138). In uveal melanoma, global SUMOylation is elevated and drives tumor growth by SUMO-modifying RB, which promotes its hyperphosphorylation/inactivation and uveal melanoma cell proliferation (139). Surprisingly, in retinoblastoma, there have been no reports showing SUMOylation of proteins, which warrants further in-depth investigation.

#### Lactylation in retinoblastoma

Protein lactylation is a reversible PTM in which lactyl groups are added to lysine residues, linking glycolytic lactate to gene regulation, signaling pathway, and tumor cell phenotypes (140, 141). Lysine lactylation has been reported to modulate transcription and cancer pathways and emerging as a potential therapeutic target (142). One group reported that alanyl-tRNA synthetase 1/2 (AARS1/2) are conserved L-lactate sensors that catalyze ATP-dependent lysine lactylation. AARS2 lactylates cyclic GMP-AMP synthase (cGAS), blocking phase separation and DNA sensing, while monocarboxylate transporter 1 (MCT1) inhibition restores innate immunity (143). Another group found that AARS1 senses lactate to drive tumor lysine lactylation, lactylates p53 to impair function, correlates with poor prognosis (144). In retinoblastoma, SUZ12-driven H3K27me3 represses Kruppel-like factor 4 (KLF4), which boosts glycolysis and lactate to elevate histone lactylation that, in turn, sustains SUZ12/H3K27me3 (145). Hence, the role of lactylation in retinoblastoma warrants further investigation to elucidate its biological functions in tumor progression (**Figure 4**).

#### Glycosylation in retinoblastoma

Protein glycosylation is a PTM in which sugars are enzymatically added to proteins, including dynamic O-GlcNAcylation where O-linked N-acetylglucosamine transferase (OGT) installs O-GlcNAc on Ser/Thr residues and O-GlcNAcase (OGA) removes it (146). Glycosylation has been confirmed to regulate signaling, transcription, and protein stability (147, 148). One group identified zebrafish-specific glycosylation sites, and reported human fibroblast growth factor 11 (FGF11) expression in stem cells and brain tumors and retinoblastoma (149). Glucose-stimulated O-GlcNAc glycosylation of YY1 disrupts its binding to the Rb, freeing YY1 to bind DNA (150). In HeLa cells, O-GlcNAcase knockdown elevates O-GlcNAc glycosylation, increasing RB phosphorylation, disturbing cyclin/CDK1 timing and spindle integrity (151). E2F1 binds and represses the OGT and OGA (MGEA5) promoters to lower protein O-GlcNAcylation, and this suppression requires RB1, as RB1-deficient cells show elevated OGT/MGEA5 and lose E2F1-mediated control (152). Neuro-1 identifies human contactin, a 135-kDa GPI-anchored glycoprotein with multiple N-linked glycosylation sites, highly expressed as multiple transcripts in retinoblastoma (153). Elf-1 undergoes phosphorylation and O-linked glycosylation, generating 98-kDa nuclear and 80-kDa cytoplasmic forms. pRB preferentially binds 80-kDa Elf-1, whereas 98-kDa conversion weakens binding, promoting nuclear migration. Hence, pRB retains Elf-1 in the cytoplasm, while phosphorylation of pRB enables Elf-1 nuclear entry and degradation (154). The role of glycosylation is required for deeper investigation in retinoblastoma (**Figure 4**).

#### Compounds target PTM in retinoblastoma

Evidence has demonstrated that numerous compounds inhibit cell proliferation, migration, invasion, metastasis, and induce cell cycle arrest and apoptosis in retinoblastoma. Moreover, multiple compounds exert their antitumor activities via regulation of protein PTMs in retinoblastoma. Here, we highlight the dozens of compounds that target protein PTMs and attenuate the development and progression of retinoblastoma (**Table 1**).

**Table 1:** Compounds targets PTMs in retinoblastoma.

Item	Model	Targets	Mechanisms	Functions	Ref
CoQ10	Y79 cells	ROS, ERK/Akt	Increases ROS, decreases pERK, pAkt, decreases VEGFA	Induces cell death, cell cycle arrest	(155)
3-MA	HXO-Rb44 cells	Akt	Decreases pAkt	Inhibits growth, enhances vincristine efficacy	(156)
Galangin	Y-79, C-33A, WERI-Rb-1, HXO-Rb44, xenograft	PTEN/Akt	Increases PTEN, decreases pAkt	Inhibits proliferation, migration, increases apoptosis	(157)
Berberine	Y79 cells	Akt, p38 MAPK	Inhibits pAkt, p-p38	Inhibits proliferation, migration, invasion	(158)
Sinomenine	WERI-Rb-1; Y79	PI3k/Akt	Inhibits p-PI3K, pAkt	Inhibits proliferation, migration, invasion, increases apoptosis	(159)
MK2206	Y79, WERI-Rb-1, HXO-RB44, NCI-H187	Akt	Reduces pAkt	Inhibits proliferation, invasion	(160)
Curcumin	Y79	JNK/p38 MAPK	Reduces pJNK, increases caspase-3/9	Inhibits viability, induces G1 arrest and apoptosis	(163)
BEZ235	Y79; WERI-Rb-1; Chx10-Cre; Rb^Lox/Lox^; p107^−/−^; Pten^Lox/Lox^ mouse model	PI3K/mTOR	Inhibits pFOXO, pS6	Induces apoptosis, inhibits tumor growth	(164)
Pentoxifylline	Y79 cells	NF-κB	Reduces p-IκBα; increases Bax/Bad/Bak	Enhances apoptosis; improves antitumor efficacy of carboplatin	(165)
TBMS II	Y79; WERI-Rb-1	TGF-β1; EGFR	Reduces pEGFR, inhibits oxidative stress	Inhibits migration, invasion, metastasis	(166)
Compounds 11, 15	BALB/c nude mice	STAT3	Reduces pSTAT3; decreases CCND1, BCL2, MYC, MMPs, VEGFA	Inhibits cell growth	(167)
Nano-MP@PSI	WERI-Rb-1; orthotopic xenograft mouse model	MDMX	Promotes MDMX ubiquitination, degradation, activates p53/p73	Increases antitumor efficacy	(171).
GSK-J4	Y79; WERI-Rb-1; Orthotopic xenografts	PI3K/AKT/NF-κB	Inhibits PI3K/AKT/NF-κB, regulates cell cycle	Induces apoptosis; induces G2/M arrest; inhibits growth	(176)
MS-275	SK cells	HDAC	Inhibits HDAC, induces p21, increases histone acetylation	Inhibits proliferation, induces apoptosis and cell cycle arrest	(178)
Sodium butyrate	Y79 cells	HDAC	Sustains H3 acetylation	Promotes neuron-like morphology, apoptosis	(179)
TSA	Y79 cells	HDAC	Increases histone acetylation	Promotes neuron-like morphology, apoptosis	(179)
WT161	Y79; WERI-Rb-1	HDAC6	Increases AcH3/AcH4 at Bad promoter, increases Bad transcription	Increases apoptosis; suppresses growth	(180)
2-DG	Y79; WERI-Rb-1; SO-Rb50, HXO-RB44; subcutaneous xenografts; LH(BETA)T(AG) transgenic mouse.	Glycolysis	Inhibits glycolysis; blocks N-glycosylation	Inhibits tumor growth; induces apoptosis; decreases angiogenesis	(183–186).

##### Targeting protein phosphorylation

CoQ10 alone or combined with Trolox kills Y79 retinoblastoma cells by generating reactive oxygen species (ROS), collapsing mitochondrial potential, enforcing G2/M arrest, and suppressing ERK and Akt phosphorylation and VEGFA (155). 3-methyladenine (3-MA) reduces Akt phosphorylation in retinoblastoma and exhibits synergistic with chemotherapeutic drug vincristine (VCR) to inhibit retinoblastoma cells (156). Galangin suppresses retinoblastoma growth by upregulating PTEN to reduce Akt phosphorylation, thereby inhibiting proliferation and migration and inducing caspase-3–dependent apoptosis *in vitro* and in xenograft models (157). Berberine inhibits retinoblastoma cell migration and invasion by reduced Akt phosphorylation and p38 phosphorylation (158). Sinomenine dose-dependently inhibits proliferation, migration, and invasion while promoting apoptosis in WERI-RB-1 and Y79 retinoblastoma cells by reducing PI3K and AKT phosphorylation (159). Tribbles pseudokinase 3 (TRIB3) drives proliferation and invasion by increasing AKT/mTOR phosphorylation, whereas TRIB3 knockdown or the AKT inhibitor MK2206 suppresses these effects in retinoblastoma (160). Additionally, miR-513b-5p suppresses retinoblastoma growth by directly targeting TRIB1, thereby reducing AKT/mTOR/p70 phosphorylation to inhibit proliferation and clonogenicity while promoting apoptosis (161). In HXO-RB44 cells, combining oncolytic adenovirus SG600 with vincristine synergistically suppresses growth by enforcing G2/M–S arrest and reducing Akt and RB phosphorylation while increasing p53 phosphorylation (162).

In Y79 retinoblastoma cells, curcumin lowers viability, induces G1 arrest, causes mitochondrial depolarization, and triggers caspase-9/3–dependent apoptosis via phosphorylation of JNK and p38 MAPK (163). In retinoblastoma models, the dual PI3K/mTOR inhibitor BEZ235 lowers phosphorylation of AKT targets p-FOXO and p-S6 to induce apoptosis in Y79 and Weri-1 cells and *in vivo*, combining BEZ235 with topotecan/carboplatin produced stronger, tumor-selective apoptosis (164). In Y79 retinoblastoma cells, pentoxifylline inhibits IκBα phosphorylation to blunt nuclear factor kappa B (NF-κB) survival signaling and, when combined with carboplatin, amplifies mitochondrial apoptosis and pro-apoptotic gene expression, such as Bax/Bad/Bak, resulting in greater antitumor efficacy than either agent alone (165). Timosaponin B-II (TBMS II) curbs TGF-β1–driven metastatic traits in Y-79 and WERI-Rb-1 retinoblastoma cells by lowering oxidative stress and inhibiting epidermal growth factor receptor (EGFR) oxidation and phosphorylation (166). Two STAT3 inhibitors (compounds 11 and 15) suppress STAT3 phosphorylation and downstream oncogenic genes, such as cyclin D1 (CCND1), BCL2, MYC, MMPs, VEGFA, which cause inhibition of retinoblastoma growth (167).

##### Targeting protein ubiquitination

A molecular glue degrader is a small molecule that induces or stabilizes an interaction between an E3 ubiquitin ligase and a target protein, promoting target ubiquitination and proteasomal degradation (168). One study showed that SPOP inhibitors stabilize STING and act as molecular glues degrading CBX4, boosting innate immunity and immunotherapy synergy in melanoma (169). Another group reported that CDDO-Me acts as a molecular glue linking EGFR to kelch-like ECH-associated protein 1 (KEAP1), inducing K63 ubiquitination and autophagy-lysosomal EGFR degradation, thereby suppressing TNBC in xenograft and organoid models (170). A peptide-derived molecular glue, nanoengineered into a pH-responsive gold–thiol complex (Nano-MP@PSI), degrades MDMX via ubiquitination to reactivate p53/p73 and delivers potent, safe antitumor efficacy in retinoblastoma models (171).

##### Targeting protein methylation

Histone demethylase inhibitor GSK-J4 elevates H3K27me3, leading to suppressing diverse oncogenic pathways across cancer models (172). One study showed that GSK-J4 inhibits jumonji domain-containing protein 3 (JMJD3) and ubiquitously transcribed tetratricopeptide repeat X chromosome (UTX) to elevate H3K27me3, suppressing A549/H1299 proliferation, EMT, invasion, and migration via Wnt/β-catenin, reducing tumors safely *in vivo* (173). KRAS-mutant lung adenocarcinoma cells are hypersensitive to GSK-J4, which increases H3K27me3, suppresses cell-cycle genes, depletes glutamate, and induces metabolic/oxidative stress (174). Donafenib and GSK-J4 show synthetic lethality in HCC, inducing ferroptosis via enhancer–promoter activation of heme oxygenase 1 (HMOX1), increasing intracellular Fe2+ across multiple preclinical models (175). GSK-J4 has been reported to suppress retinoblastoma growth by inducing G2/M arrest and apoptosis *in vitro* and *in vivo*, mechanistically linked to inhibition of the PI3K/AKT/NF-κB signaling pathway (176).

##### Targeting protein acetylation

HDAC inhibitors block histone deacetylases, increasing histone acetylation, opening chromatin, reprogramming gene expression, and often promoting tumor cell differentiation, apoptosis, or immunogenicity (177). The HDAC inhibitor MS-27–275 rapidly increases histone acetylation, induces p21, and suppresses proliferation across pediatric solid tumor lines including retinoblastoma, producing variable G1/G2 arrest or apoptosis (178). In Y79 retinoblastoma cells, HDAC inhibitors sodium butyrate and trichostatin A (TSA) trigger transcription-dependent neuron-like morphology and apoptosis, with sustained elevations of histone H3 acetylation for butyrate and transient acetylation for TSA (179). WT161, a selective HDAC6 inhibitor, suppresses retinoblastoma growth and induces apoptosis, synergizes with cisplatin by increasing AcH3/AcH4 at the Bad promoter to elevate Bad transcription (180).

##### Targeting protein glycosylation

2-deoxy-D-glucose (2-DG) inhibits glycolysis and disrupts N-linked glycosylation by limiting mannose incorporation, inducing ER stress and impaired protein maturation (181, 182). In a retinoblastoma mouse model, subconjunctival 2-DG reprogrammed region-specific expression of angiogenesis, hypoxia, metabolism, and apoptosis genes (183). In a retinoblastoma mouse model, periocular 2-DG reduced tumor burden and hypoxia dose-dependently, and synergized with carboplatin to enhance efficacy (184). In retinoblastoma, NAT10 elevates ac4C to stabilize 6-phosphofructo-2-kinase/fructose-2,6-bisphosphatase 3 (PFKFB3) mRNA, boosting glycolysis and tumor growth. Moreover, 2-DG counteracts NAT10-driven glycolytic activation (185). 2-DG inhibits angiogenesis by blocking N-linked glycosylation, inducing unfolded protein response (UPR)/CCAAT-enhancer-binding protein homologous protein (CHOP) apoptosis, suppressing endothelial growth and migration and neovascularization in retinoblastoma (186). Hence, targeting protein glycosylation is hopeful for improving retinoblastoma treatment.

#### PTMs of Rb and immunotherapy

Retinoblastoma treatment emphasizes local drug delivery (intravitreal chemotherapy, ophthalmic artery chemosurgery) for eye salvage, while novel agents, oncolytic viruses, and immunotherapy aim to improve intraocular and metastatic outcomes (187). Because chemotherapy for retinoblastoma can fail due to resistance and metastasis, emerging immunotherapy strategies target tumor cells and the microenvironment via disialoganglioside GD2, programmed cell death protein 1/programmed death-ligand 1 (PD-1/PD-L1), B7 homolog 3 (B7H3), epithelial cell adhesion molecule (EpCAM), or spleen tyrosine kinase (SYK) using chimeric antigen receptor T cell (CAR-T), bispecific antibodies, and engineered dendritic cells (188). Low-toxicity immunotherapy targets GD2, with dinutuximab, GD2 CAR-T, vaccines, and nanoparticles showing promise (189). In the following paragraphs, we will discuss the role of RB and its PTMs in immunotherapy.

##### Immunotherapy in retinoblastoma

In RB-Y79 retinoblastoma cell line, low-dose carboplatin primes tumors for DC-Ag (dendritic cells pulsed with tumor antigens)–CIK (cytokine-induced killer cells) immunotherapy, enhancing engagement and apoptosis (190, 191). In retinoblastoma, EpCAM+ cells co-express stem markers and show enhanced invasion/neurospheres. EpCAM and CD3 antibodies redirect T cells, suppressing tumor growth and inducing cytokines, supporting immunotherapy (192). In RB, SYK-presenting dendritic cells prime CTLs that selectively kill SYK-positive, including carboplatin-resistant RB-Y79 cells, supporting SYK-targeted adoptive immunotherapy for chemoresistant retinoblastoma (193). Retinoblastoma tumors overexpress B7H3 with spatial heterogeneity and low T-cell infiltration, supporting B7H3-targeted immunotherapy, particularly in poorly differentiated noninvasive retinoblastoma (194). In 94 retinoblastoma samples, B7-H3 and GD2 were most prevalent, while CD171 and PD-L1 were rare, supporting antigen-guided CAR-T immunotherapy strategies (195). In 262 retinoblastoma cases, PD-1/PD-L1 differ between primary and chemoreduced tumors and predict survival, supporting immune checkpoint–based immunotherapy for chemoresistant disease (196).

In retinoblastoma, immune gene signatures stratify tumors into high-ICI and metastatic-prone groups. DNA hypermethylation links to immune regulation, informing immunotherapy target discovery (197). In retinoblastoma, tuftsin-loaded carbonized MOF nanoparticles enable PA/MR imaging, photothermal ablation, and macrophage-activating immunotherapy, with good biosafety (198). Phase I immunotherapy racotumomab vaccine targeting NeuGcGM3 was well tolerated and induced IgM/IgG immune responses in retinoblastoma patients (199). Preclinical retinoblastoma immunotherapy shows CD171 and GD2 CAR-T cells robustly activate and kill RB lines. Sequential dual-targeting improves killing and mitigates antigen loss (200). In four relapsed metastatic retinoblastoma patients, dinutuximab beta showed responses and durable remissions. Anti-GD2 and anti-CD276 mediated ADCC (anti-GD2 antibody-dependent cell-mediated cytotoxicity) and CDC (complement-dependent cytotoxicity) killing *in vitro* (201). Therefore, immunotherapy could be a promising strategy for retinoblastoma treatment.

##### Rb PTMs involve in immunotherapy

In TNBC PDXs, irinotecan response was predicted by BRCAness, high SLFN11, and RB1 loss. Moreover, ataxia telangiectasia and Rad3-related (ATR) inhibition sensitized resistant models, linking DNA repair checkpoints to immune-relevant damage (202). Valproic acid, an HDAC inhibitor, reduces polymorphonuclear myeloid-derived suppressor cell (PMN-MDSC) abundance and suppressive function via IL-4Rα/arginase, PD-L1/TLR4, and RB1 derepression, enhancing immune responsiveness to immune checkpoint blockade (ICB) (203). In 95 TNBC, multimodal profiling linked RB1 mRNA/protein to RB function, aligned PD-L1 immune phenotypes, refined LAR subgroups, and found human epidermal growth factor receptor 2 (HER2)-enriched tumors with improved survival (204). In LUAD, RB1 mutation increases PARPi sensitivity by inducing genomic instability and cytosolic DNA, activating cGAS/STING, elevating C-C motif chemokine ligand 5/C-X-C motif chemokine ligand 10 (CCL5/CXCL10), and promoting immune infiltration (205). In radioresistant glioma stem cells, AMPK-driven RB phosphorylation disrupts KDM5A control of CD47, boosting epigenetic CD47 and immune-evasive M2 macrophages after radiotherapy (206). Protein phosphatase 2A (PP2A) loss converts microsatellite stable (MSS) to microsatellite instable (MSI) by RB phosphorylation–E2F– DNA methyltransferase 3 alpha/3 beta (DNMT3A/3B)–mediated DNA methylation and HDAC2 phosphorylation–driven MLH1 silencing, inducing neoantigens, T-cell infiltration, and ICB sensitivity (207). Hyperphosphorylated RB (S249/T252) binds NF-κB p65 to repress NF-κB–driven PD-L1. RB loss or CDK4/6 inhibition elevates PD-L1, while a phosphomimetic peptide enhances radiotherapy efficacy (208). It is important to mention that the link between PTMs and immune regulation is supported by existing evidence. Further exploration is necessary to determine the critical role of RB PTMs in immunotherapy.

## Conclusions and perspectives

In conclusion, PTMs orchestrate retinoblastoma initiation, progression, metabolic adaptation, and therapy resistance by rewiring RB-centered signaling and chromatin control. Mapping PTM crosstalk and exploiting druggable enzymes may enable rational combinations with emerging immunotherapies (GD2/CAR-T, PD-1/PD-L1, B7H3) to improve durable eye-salvage and metastatic control. Several critical questions remain to be addressed. For example, why are PTMs important in retinoblastoma? Which PTMs have been most convincingly validated in retinoblastoma? Which PTM-regulating enzymes are the most druggable? Recently, several newly identified PTMs, such as pyruvoylation and vitamin C-related modifications, have been reported (209, 210). However, whether these modifications occur in retinoblastoma remains unknown and has not yet been explored. In addition, engineered mouse models and patient-derived samples will be essential for further elucidating the roles of PTMs in retinoblastoma. Several perspectives should be mentioned. First, construct an RB-centric PTM atlas with clinical-state resolution in retinoblastoma is pivotal. Build a retinoblastoma PTM atlas centered on the RB1–E2F checkpoint, integrating phospho-/acetyl-/ubiquitin-/methyl-proteomics with bulk and single-cell transcriptomics to resolve cell-state–specific circuitry. Because PTMs are dynamic and reversible, sampling under therapy and stress conditions, such as DNA damage, hypoxia, immune responses should identify cooperative pathways beyond RB phosphorylation alone. Second, protein SUMOylation remains essentially unreported in retinoblastoma, while lactylation and glycosylation are only emerging. It is necessary to explore SUMO/lactyl/O-GlcNAc modification in retinoblastoma and determine how these PTMs reshape chromatin, metabolism, and immune sensing in microenvironmental niches, and define new substrates. Third, it is required to develop PTM-targeting–immunotherapy combinations with mechanistic endpoints. Retinoblastoma immunotherapies targeting GD2, PD-1/PD-L1, B7H3, EpCAM or SYK (CAR-T, bispecifics, engineered dendritic cells) are advancing, yet resistance persists. Prioritize PTM-directed therapeutics as combinatorial partners for immunotherapy could be useful. HDAC inhibitors, kinase-pathway modulators, and ubiquitin-pathway degraders could be screened for effects on antigen density and PD-L1 regulation to rationally design synergy. Fourth, it is important to move toward biomarker-guided, globally applicable translation. Prospective registries should pair PTM biomarkers with treatment response and toxicity, enabling risk-adapted escalation.
